# The dopaminergic control of Cushing’s syndrome

**DOI:** 10.1007/s40618-021-01661-x

**Published:** 2022-04-23

**Authors:** R. Pivonello, C. Pivonello, C. Simeoli, M. C. De Martino, A. Colao

**Affiliations:** 1 Dipartimento Di Medicina Clinica E Chirurgia, Sezione Di Endocrinologia, Università Federico II Di Napoli, Naples, Italy; 2 UNESCO Chair for Health Education and Sustainable Development, Federico II University, Naples, Italy

**Keywords:** Cushing’s syndrome, Cushing’s disease, Dopaminergic system, Dopamine, Cabergoline

## Abstract

Cushing’s Syndrome (CS), or chronic endogenous hypercortisolism, is a rare and serious disease due to corticotroph pituitary (Cushing’s disease, CD) and extra-pituitary (ectopic CS) tumours overproducing ACTH, or cortisol-secreting adrenal tumours or lesions (adrenal CS). The first-line treatment for CS is represented by the surgical removal of the responsible tumour, but surgery might be unfeasible or ineffective and medical treatment can be required in a relevant percentage of patients with CS, especially CD and ectopic CS. Corticotroph pituitary and extra-pituitary tumours, as well as adrenal tumours and lesions responsible for CS express dopamine receptors (DRs), which have been found to mediate inhibition of hormone secretion and/or cell proliferation in experimental setting, suggesting that dopaminergic system, particularly DRs, might represent a target for the treatment of CS. Dopamine agonists (DAs), particularly cabergoline (CAB), are currently used as off-label treatment for CD, the most common form of CS, demonstrating efficacy in controlling hormone secretion and tumour growth in a relevant number of cases, with the improvement of clinical picture, and displaying good safety profile. Therefore, CAB may be considered a reasonable alternative treatment for persistent or recurrent CD after pituitary surgery failure, but occasionally also before pituitary surgery, as adjuvant treatment, or even instead of pituitary surgery as first-line treatment in case of surgery contraindications or refusal. A certain beneficial effect of CAB has been also reported in ectopic CS. However, the role of DAs in the clinical management of the different types of CS requires further evaluations.

## Introduction

Cushing’s Syndrome (CS), or chronic endogenous hypercortisolism, is a rare endocrine disease associated with increased morbidity and mortality, which is consequence of several chronic comorbidities and systemic complications [1, 2]. CS can be caused by a corticotroph pituitary tumour overproducing ACTH [Cushing’s disease (CD)] in about 70%, an adrenal tumour or bilateral adrenal hyperplasia or dysplasia overproducing cortisol (adrenal CS) in about 20%, and an extra-pituitary tumour overproducing ACTH or, rarely, corticotrophin-releasing hormone (CRH) (ectopic CS) in about 10% of cases [1, 2]. The first-line treatment for CS is the surgical removal of the responsible tumour. However, especially in ACTH-dependent forms of CS (CD and ectopic CS), surgery might be unfeasible or ineffective, determining persistence or recurrence of cortisol excess [3]. Medical treatment can be required to manage chronic hypercortisolism, preventing systemic complications and normalizing mortality. However, the medical agents currently available for controlling hypercortisolism and targeting corticotroph pituitary tumours, adrenal glands, or glucocorticoid receptors are effective in subsets of patients with CS [3, 4]. In this context, the expression of dopamine receptors (DRs) in corticotroph pituitary and extra-pituitary tumours, as well as in adrenal lesions, causing CS, represents a potential target for the treatment of the different forms of CS with dopaminergic drugs, particularly dopamine agonists (DAs).

Dopamine is a catecholamine acting as either central neurotransmitter or peripheral hormone, regulating several physiological processes, through the binding to DRs, a class of seven transmembrane domains G protein coupled receptors, consisting of five different subtypes (D1-D5 receptors) [5–9]. In the hypothalamus-pituitary system, dopamine regulates the pituitary hormone production, either in physiological or pathological conditions [6, 10]. In physiological conditions, dopamine mainly inhibits the prolactin (PRL) secretion from lactotroph cells [5, 11], but it also regulates hormone secretion from non-lactotroph cells, mainly through the activation of D2 receptor [5, 12, 13], whereas, in pathological conditions, the expression of DRs in several types of hormone-secreting, or functioning, as well as non-functioning, pituitary tumours represents the prerequisite for the employment of DAs, mainly bromocriptine (BRC) and cabergoline (CAB), in the management of different diseases associated with the pituitary tumours [14–17].

The expression of DRs in the hypothalamus–pituitary–adrenal (HPA) axis and in the diffuse neuroendocrine system in physiological or pathological conditions suggests a potential role of the dopaminergic system in the regulation of HPA axis and diffuse neuroendocrine system and a potential role of the dopaminergic drugs in the management of CS. Indeed, normal pituitary corticotroph cells [12], extra-pituitary neuroendocrine cells [9], and cortisol-producing adrenal cells [18] express DRs, suggesting a potential physiological role of DRs in the regulation of HPA axis and diffuse neuroendocrine system. On the other hand, the tumours responsible for CS, including corticotroph pituitary tumours [19–23], extra-pituitary neuroendocrine tumours [24], and cortisol-producing adrenal tumours [18], have been described to express DRs, mainly D2 receptors, which represent potential targets for the treatment of CS [18, 19, 23, 24].

CAB has been experimented in the medical treatment of CD [3], and displayed, according with a recent meta-analysis, a remission rate of 34% (20–40%) in patients treated for a period ranging from 1 to 105 months [25, 26]. CAB treatment was associated with an improvement of clinical syndrome and complications with a good safety [25, 27–32]. CAB has been also evaluated in combination with steroidogenesis inhibitors, showing a remission rate of 68.8% (range 55.6–78.6%) in CD patients unsuccessfully treated by pituitary surgery [29, 30, 32], and in combination with the somatostatin receptor ligand (SRL) pasireotide, showing an additional remission rate of 33.3% of patients with CD [33, 34]. No studies specifically reported data on the efficacy and safety of CAB on adrenal CS, whereas case reports and small series have shown a beneficial effect of CAB in monotherapy [24, 35, 36], in therapeutic sequence or in combination with steroidogenesis inhibitors [36] or SRLs [37–39] in patients with ectopic CS. Few cases have also suggested the hypothesis that CAB may be useful in patients with Nelson's syndrome, or corticotroph tumour progression, induced by bilateral adrenalectomy in patients with CD [40, 41]. However, further controlled prospective studies on larger populations of patients are needed to better evaluate the clinical advantages, in terms of efficacy and safety, of the treatment with dopaminergic drugs in the management of the different forms and consequences of CS.

The present review summarizes the current preclinical and clinical data on the role of dopamine and DAs in CS.

## Dopamine and dopamine receptor structure and function

Dopamine is a catecholamine acting both as neurotransmitter in the central nervous system as well as a hormone in the endocrine system. Dopamine synthesis starts from l-tyrosine and follows two steps; in the first step, tyrosine hydroxylase catalyses the production of l-DOPA that, in the second step, through the action of DOPA decarboxylase, is converted in dopamine [8]. In the central nervous system, dopamine is produced by dopaminergic neurons mainly belonging to four different areas, including ventral tegmental area of the midbrain, where it is implicated in the regulation of motivation and pleasure, reward, cognition, hunger and satiety; pars compacta of substantia nigra, where it is involved in the regulation of locomotion and learning; retina and olfactory bulb, where it is involved in the regulation of circadian rhythm; and arcuate nucleus of the hypothalamus, where it is implicated in the regulation of lactation and reproduction through the PRL secretion by the pituitary gland [8, 42]. Indeed, more recently, the dopaminergic system has emerged as modulator of circadian clock entrainment in the central nervous system, where dopamine presents a circadian-like activity, which regulates and is regulated by the circadian clock machinery [42]. In the endocrine system, the cells of adrenal medulla and diffuse neuroendocrine system represent the main peripheral sources of dopamine, which is involved in several actions mainly including the regulation of gastrointestinal motility and blood pressure, as well as the hormone production. Dopamine is released through secretory vesicles, as neurotransmitter, in the synaptic cleft by presynaptic neurons where it activates paracrine functions in postsynaptic neurons, whereas, as hormone, in the extracellular space or in the bloodstream where it activates autocrine and paracrine functions at local target organs as well as more classical endocrine functions at distant target organs [43].

**Fig. 1:**
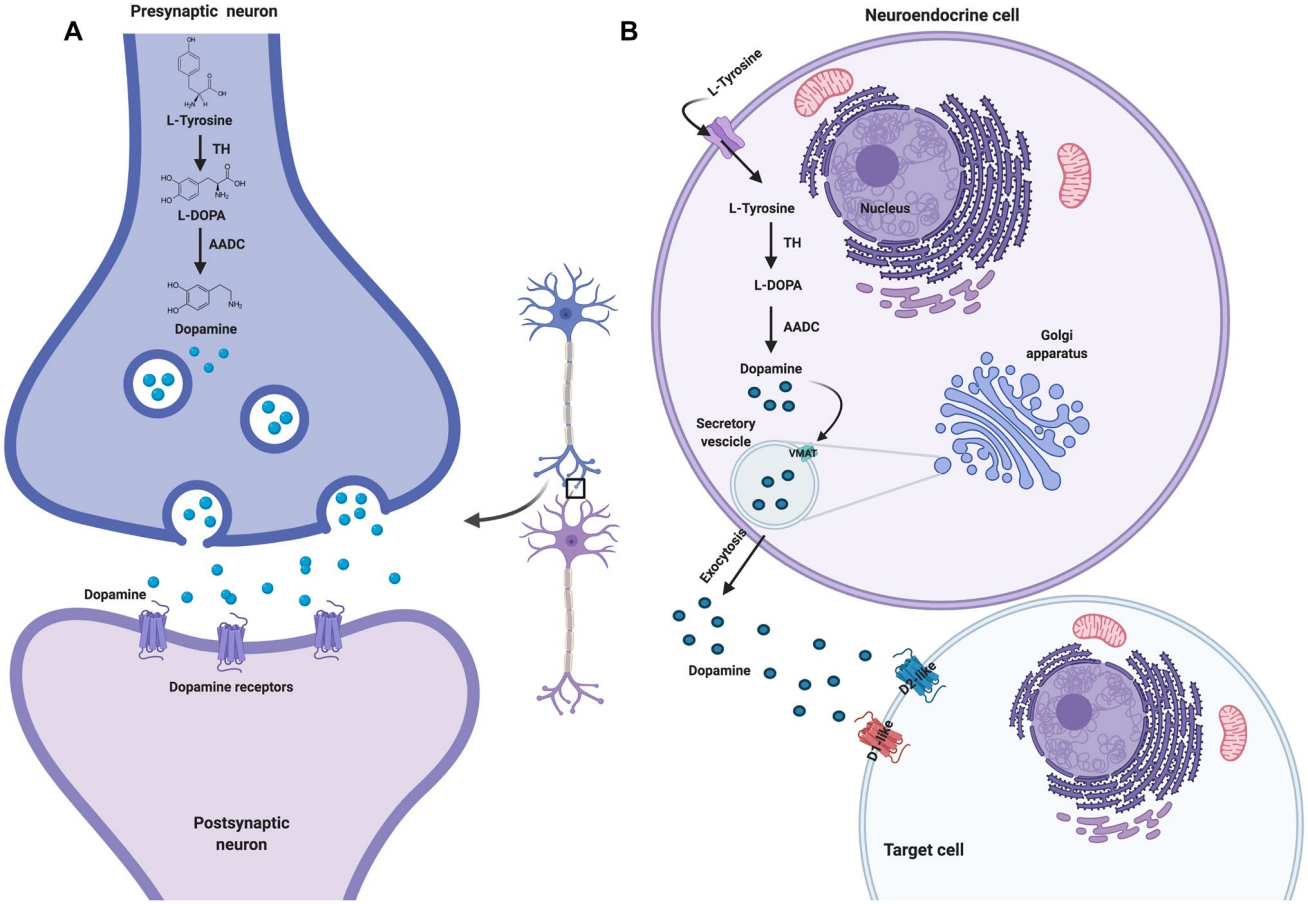
Dopamine synthesis and release by neurons and neuroendocrine cells. Dopamine synthesis starts from l-tyrosine. In the first step, tyrosine hydroxylase (TH) catalyses the production of l-DOPA that, in the second step, is converted in dopamine by the action of the DOPA decarboxylase (AADC). After the synthesis, vesicular monoamine transporter (VMAT) transports dopamine from the cytoplasmic space into secretory vesicles that by exocytosis induce dopamine release in the synaptic cleft, extracellular space or in bloodstream. After the release, dopamine can bind and activate dopamine receptors (DRs) (Created with BioRender.com)

Figure 1 shows a schematic representation of the dopamine synthesis and release in neurons and neuroendocrine cells.

Dopamine mediates its physiological actions by binding to five different DRs localized on the membrane of the target cells with different affinity ranging from nanomolar to micromolar range [7, 8, 43]. DRs are distinct in two major types of seven transmembrane domain G protein coupled receptors, classified on the basis of their structural, functional and pharmacological properties: the excitatory D1-like receptors, comprising D1 and D5 receptors, and the inhibitory D2-like receptors, comprising D2, D3 and D4 receptors [5–7]. The five DRs are structurally characterized by an extracellular NH_2_-terminal stretch, seven transmembrane domains, three extracellular and three intracellular loops and an intracellular COOH-terminal tail [5, 6]. The NH_2_-terminal stretch has a similar number of amino acids in the entire series of DRs and contains N-glycosylation sites that ensure the structure and integration of the receptor into the cell membrane, with a consequential functional role in the intracellular receptor trafficking [44]. The COOH-terminal tail is seven times longer in D1-like than in D2-like receptors and contains cysteine residues that, together with cysteine residues present in the second and third extracellular loops, form disulphide bridges involved in the stabilization of the receptor structure and anchorage of the receptor to the cell membrane, as well as serine and threonine residues whose phosphorylation allows the activation of intracellular signal transduction pathways [5, 6]. D1-like receptors are encoded by genes not interrupted by introns, whereas D2-like receptors are encoded by genes interrupted by introns [5–7]. The genetic sequence organization in D2-like receptors provides the basis for splicing variants generation; D2 receptor exists in two different isoforms, D2 short (D2S) and D2 long (D2L), differing for the presence or absence of 29 amino acids in the third intracellular loop, and displaying distinct physiological and pharmacological properties [5, 6, 45, 46]. Splicing variants have also been described for D3 receptor, despite essentially encoding for non-functional isoforms [5, 6, 47]. Polymorphic variants have been identified for D4 receptor, with the different isoforms characterized by repeated sequences in the coding region of the third intracellular loop and displaying different physiological and pharmacological properties [5, 6, 48]. Dopamine or DAs, binding to DRs, induces the activation of G protein subtypes, which transduces the signal to activate different effector systems in the various target cells and may differ in dependence of the dopamine role in physiological conditions as well as the DAs actions in pathological conditions [6, 7].

The physiological role of dopamine has been better characterized in the lactotroph cells of the pituitary gland. The most relevant physiological mechanism mediated by DRs, according to studies in rodent and human cell models, is the regulation of hormone secretion, through the modulation of cyclic AMP (cAMP) pathway. Based on the increasing evidence of the last decades, D1-like receptors are coupled with stimulatory G proteins (Gα_s_) to induce adenylyl cyclase (AC) activity, cAMP accumulation and protein kinase A (PKA) activation and to stimulate calcium (Ca^2+^) release from the intracellular storage, whereas D2-like receptors are coupled with inhibitory G proteins (Gα_i/o_) to suppress AC activity, cAMP accumulation and PKA activation and to inhibit Ca^2+^ release from the intracellular storage [5, 6, 49]. Notably, Gα_s_ and Gα_i/o_ also modulate potassium (K^+^) channels, which mediate the regulation of hormone secretion, acting in opposite manner compared to Ca^2+^ channels [7]. Finally, Gα_s_ and Gα_i/o_ are also deputed to modulate, through G protein coupled receptor kinases (GRKs), protein kinase C (PKC) and β-arrestins, and the control mechanism of receptor internalization, the functional receptor sensitization or desensitization to dopamine and DAs [5, 6, 49]. Beside the regulation of hormone secretion, in lactotroph cells, DRs also mediate the regulation of the complex physiological mechanism of cell homeostasis, a condition of biological and molecular steady state, which is useful to prevent the deregulation of dopamine signalling pathway and needful not only to limit the PRL synthesis and secretion, but also the lactotroph cell growth and proliferation, in order to restrict the development of lactotroph hyperplasia, or lactotroph differentiation and expansion with a consequent formation of lactotroph tumours [50–55]. Studies on rodent lactotroph cell models revealed that D2 receptor, and particularly both D2S and D2L, trigger cAMP-independent pathways, such as mitogen-activated protein kinase (MAPK) pathway, including ERK1/2, and phosphatidylinositol 3-kinase (PI3K)/protein kinase B (PKB) pathway, which is known as AKT [50–55]. Indeed, new insights have been emerged in molecular mechanism regulation by D2 receptor, and particularly D2S and D2L, in rodent lactotroph cell lines, demonstrating that the activation of D2S stimulates, whereas the activation of D2L inhibits, MAPK and AKT, playing a critical role in cell growth, proliferation and differentiation, as well as hormone synthesis and release [51, 52]. The control of D2L/D2S ratio, with specific crosstalk and balance between MAPK and AKT pathways, are required to maintain lactotroph cell homeostasis and to prevent pathological lactotroph expansion. Indeed, D2L null mice display an upregulation whereas D2S null mice a downregulation of MAPK and AKT [52]. In physiological condition, the presence of one D2 receptor isoform is sufficient to control cell proliferation and maintain normal hormone production, while in the pathological condition following the simultaneous ablation of both D2 receptor isoforms, such as in D2R null mice models, an opposite outcome on MAPK and AKT signalling pathways is observed, with inhibition of MAPK activity and stimulation of AKT activity, resulting in loss of control of cell proliferation and consequent lactotroph cell hyperplasia and hormone hypersecretion [52]. On the other hand, in the pathological condition following oestradiol exposure, which induces the secretion of different pituitary growth factors involved in pituitary cell proliferation, the expression of one D2 receptor isoform is no longer sufficient to control lactotroph cell homeostasis; indeed, the interference of oestradiol with the function of D2 receptor isoforms induces a decrease of MAPK activity in D2L null and an increase of AKT activity in D2S null mice models, with a surprising decrease of AKT but not MAPK activity in D2R null mice models [52]. The regulation of the cell growth and proliferation by dopamine has been also confirmed in pituitary tumours where the activation of D2 receptor, especially D2S, leads to the activation of the MAPK pathway, c-Jun N-terminal kinase and early growth response protein 1 and the induction of apoptosis, consequently leading to the suppression of cell growth and proliferation in rodent lactotroph and somatotroph cell lines as well as in primary cultures of human non-functioning pituitary tumours [51, 55–57]. Interestingly, it has been recently demonstrated that the activation of D5 receptor, in several pituitary tumours, inhibits tumour growth and induces autophagic cell death by inhibiting PI3K/AKT/mTOR pathway and by increasing reactive oxygen species formation in rodent lactotroph and somatotroph tumour cells [58, 59].

**Fig. 2:**
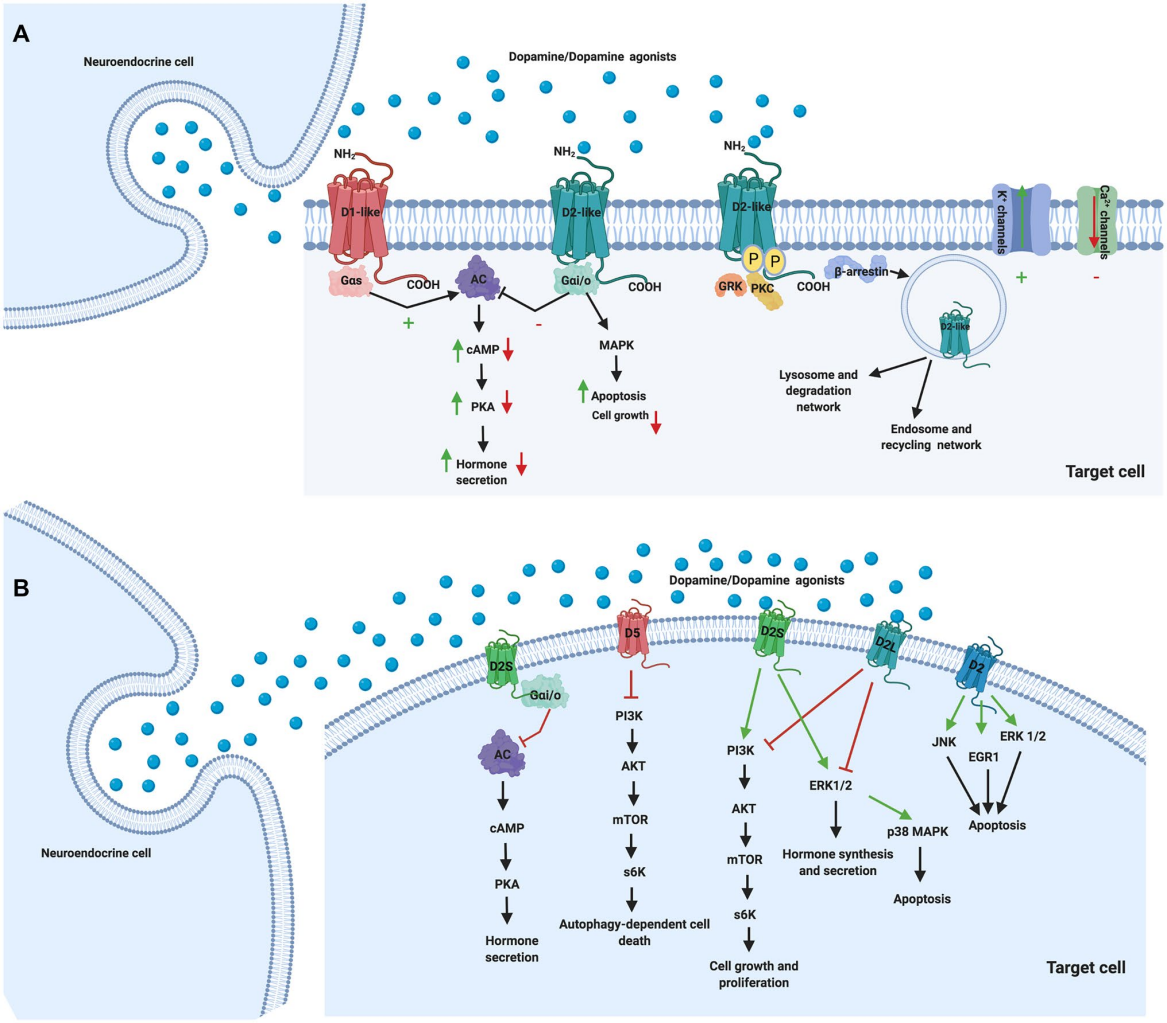
**A** Main molecular signalling pathways induced by dopamine and DAs. Dopamine (or DAs) binding to D1-like and D2-like receptors, via interaction with G_αs_ or G_αi/o_ proteins, stimulates or inhibits adenylyl cyclase (AC) activity, cyclic AMP (cAMP) and protein kinase A (PKA), modulates K^+^ channels and Ca^2+^ currents in an opposite manner, regulating hormone secretive effects in neuroendocrine cells. D2-like receptors activation modulates mitogen-activated protein kinases (MAPKs) increasing apoptosis and inhibiting cell growth. Moreover, D2-like receptors are involved in receptor desensitization and degradation signals recruiting β-arrestin via GRKs and protein kinase C (PKC) activation. **B** Main molecular signalling pathways specifically triggered by D2 receptor (D2S and D2L) and D5 receptors. D2S activation induces hormone secretion inhibition by the suppression of AC activity, cAMP and PKA. However, in lactotroph cells, extracellular-regulated kinase (ERK) and phosphatidylinositol 3-kinase (PI3K) pathways are oppositely regulated by D2S and D2L, with D2S stimulating both and D2L inhibiting both pathways involved in cell growth, proliferation, and lactotroph homeostasis. D2 receptor is also involved in apoptotic process through the activation of ERK1/2, c-Jun N-terminal kinase (JNK) and early growth response protein 1 (EGR1), and specifically by D2S via p38 MAPK. Cell proliferation and growth are also inhibited by the induction of autophagic dependent cell death triggered by D5 receptor via PI3K/AKT/mTOR pathway. (Created with BioRender.com)

Fig. 2A shows a schematic representation of the main intracellular mechanisms induced by D1-like and D2-like receptors regulating hormone secretion and cell proliferation and apoptosis. Figure 2B reports the specific intracellular mechanisms triggered by D2 receptor (D2S and D2L) and D5 receptors upon binding of dopamine and DAs.

## Dopamine receptor expression in normal HPA axis and diffuse neuroendocrine system

DRs are widely expressed in the organs that compose the HPA axis of the endocrine system, including hypothalamus, as well as pituitary gland and adrenal glands, where dopaminergic pathways are involved in the regulation of hormone synthesis and secretion, as well as in the diffuse neuroendocrine system, composed by neuroendocrine cells present in the great majority of organs and systems, including upper respiratory tract and lungs, thyroid gland, gastrointestinal system, gallbladder, and pancreas, where dopamine regulates several specific physiological processes.

DRs expression has been demonstrated in the normal hypothalamus. In humans and rodents, DRs are expressed in different hypothalamic cell populations; in particular, D2 receptors is expressed in subpopulations of neurons expressing thyrotropin or somatostatin [6, 10]. Importantly, DRs expression has been clearly demonstrated in the normal pituitary gland. In rodents and humans, the pituitary gland is anatomically composed by two major lobes: the anterior lobe, which has an epithelial structure, and the posterior lobe, which has a neuronal structure, separated by the “pars intermedia”, which has a connective structure with interspersed basophilic cells. The anterior lobe harbours several different types of secreting endocrine cells, accounting for 60–70% of acidophil cells (lactotroph and somatotroph cells) and 30–40% of basophilic cells (thyrotrophs, corticotroph and gonadotroph cells). In rodents and humans, D2 receptor is expressed in cells of the anterior lobe, mainly in lactotrophs [5, 11], but also in non-lactotroph cell populations, including somatotrophs, thyrotrophs and gonadotrophs, and, in humans, also in corticotrophs, where D2 receptor has been suggested to have a regulatory role of hormone secretion [5, 23, 60–62]. In rodents and humans, the pituitary posterior lobe hold the dopaminergic projections of the substantia nigra, ventral tegmental area and preoptic periventricular hypothalamic region that reach the neurohypophyseal magnocellular neurons expressing DRs, particularly D2 and D4 receptors, responsible for the regulation of the oxytocin and vasopressin release [63–65], although the exact physiological role of dopaminergic inputs to the neurohypophyseal magnocellular neurons have not been well-characterised. In rodents, the pars intermedia, or intermediate lobe, is well defined and composed by densely arranged basophilic cells separated into lobules by strands of connective tissue [66]. Conversely, in humans, the pars intermedia is clearly distinct during foetal life but became scarcely defined in adult life, where it is represented by a group of colloid-filled cysts interspersed in the portion of posterior lobe adjacent to the anterior lobe [12, 67]. In particular, in humans, the pars intermedia consists of a subgroup of specialized corticotroph cells, the melanotroph cells, which release melanocyte-stimulating hormone (MSH) and ACTH, and that surround the colloid-filled cysts. Moreover, a group of basophilic cells of the anterior lobe, belonging to the corticotroph cells and almost exclusively releasing ACTH, can infiltrate the residual pars intermedia and can be found in direct contact with the colloid-filled cysts, leaning into the posterior lobe [12]; the totality of the area, including the canonical pars intermedia and the basophilic corticotroph cells belonged to the anterior lobe, but infiltrated among the cysts and adjacent to neural cells of posterior lobe, has been identified as “intermediate zone” by Pivonello and co-workers [12]. In the intermediate zone, D2 receptor has been found to be strongly expressed in corticotroph cells of canonical pars intermedia lining the colloid-filled cysts, presumably representing the remnant melanotroph cells of the residual pars intermedia and, with a variable expression ranging from weak to strong, in the anterior lobe corticotroph cells located between the cysts [12]. Moreover, the evidence that D2 receptor is expressed more intensively in the special corticotroph cells of the “intermediate zone” rather than in the typical corticotroph cells of the anterior lobe suggested the existence of different corticotroph cell populations that could potentially spread in human normal pituitary gland with different features, behaviour and role; this evidence is suggestive of a relevant role of DRs in the special corticotroph cells of the pars intermedia and probably of the intermediate zone, with less relevant role in the typical corticotroph cell of the anterior lobe [12]. Notably, D2 receptor is also moderately expressed in the fibers and in neural cell bodies of the posterior lobe of the pituitary gland [12].

**Fig. 3:**
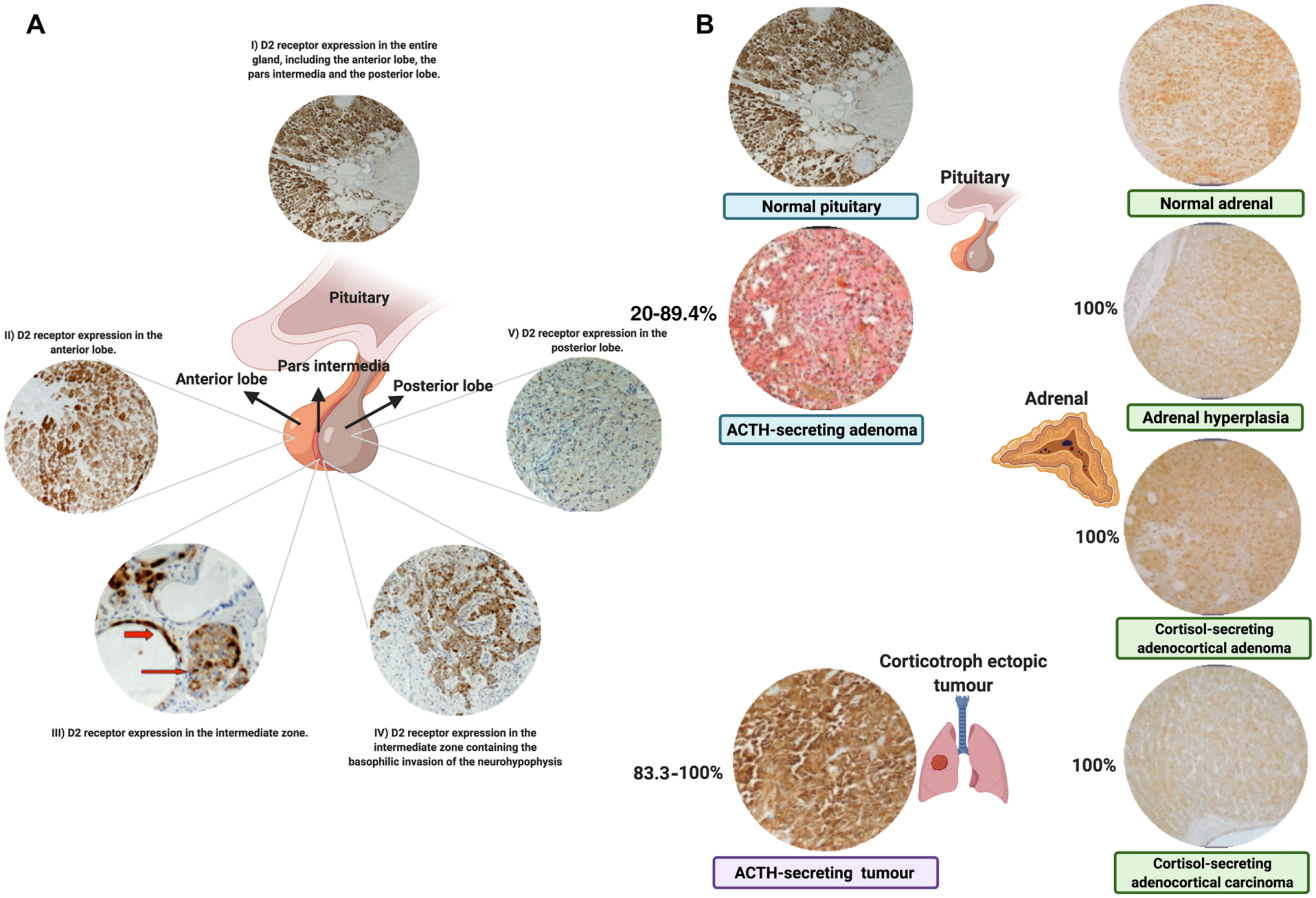
**A** D2 receptor expression in **I** the entire gland, with magnification on **II** anterior lobe, **III** and **IV** “pars intermedia” and **V** posterior lobe of human normal pituitary. **III** Red arrows: D2 receptor expression in cells lining the colloid-filled cysts belonging to the pars intermedia and in cell cluster belonging to the anterior lobe and located within the cysts adjacent to the neural posterior lobe. **IV** The picture focuses on the intermediate zone between the anterior and the posterior lobe containing the basophilic invasion of the neurohypophysis. The picture shows a strong homogeneous and diffused expression of D2 receptor in the corticotroph cell population invading the neurohypophysis. **B** Exemplary pictures of D2 receptor protein expression and the relative rate of expression in normal pituitary and adrenal glands, corticotroph pituitary tumours, adrenocortical tumours and in a case of ectopic corticotroph tumours. The different shades of brown highlight the immunostaining for D2 receptor with a specific polyclonal D2 receptor antibody. (Created with BioRender.com)

Figure 3A shows the expression of D2 receptor in the anterior lobe, pars intermedia and posterior lobe of human pituitary gland.

The expression and function of DRs have been also investigated in the human and rodent normal adrenal gland with the same pattern of distribution. D1-like and D2-like receptors are expressed in the normal adrenal gland. In particular, D2 and D4 receptor are localized in the three areas of the adrenal cortex and in the adrenal medulla where DRs mediate the dopamine regulation of aldosterone, cortisol, sex hormones, and catecholamines secretion, respectively [18, 68–72]. Nevertheless, D2 receptor is mainly expressed in the zona glomerulosa and reticularis of the adrenal cortex [18, 70], suggesting a pivotal role in the control of aldosterone and sex hormone secretion.

The expression and function of DRs has been also investigated in the human and rodent neuroendocrine cells of the diffuse neuroendocrine system, in which D1-like and D2-like receptors have been demonstrated to be expressed and involved in the regulation of hormone secretion and cell differentiation, together with angiogenesis, and indirectly in the control and protection of gastrointestinal function and control of renal vascular system [6, 73].

### Dopaminergic drugs

Several selective compounds have been developed as DAs displaying different pharmacological properties including the binding selectivity for the different DRs. DAs may be divided into ergot-derivatives, which include BRC, CAB, pergolide and lisuride, and non-ergot-derivatives, which mainly include quinagolide, and they represent a useful medical therapy in the management of Parkinson’s disease [74]. The discovery of the dopamine inhibitory effect on PRL secretion and lactotroph tumour growth more recently led to an increasing use of DAs in the endocrinological field for the management of disorders associated with hyperprolactinemia [16]. BRC and CAB, are the most commonly used DAs in the treatment of endocrine diseases, whereas pergolide, quinagolide and lisuride have been sporadically used in the endocrine field [75].

Table 1 reports the pharmacological properties, particularly the binding affinities to cloned human DRs, of the different available DAs.

**Table 1:** Pharmacological properties of the different available DAs: binding affinities to cloned human DRs

Dopamine agonists	Receptor subtypes (binding affinity)					
	D1 (Ki, nM)	D2S (Ki, nM)	D2L (Ki, nM)	D3 (Ki, nM)	D4 (Ki, nM)	D5 (Ki, nM)
Bromocriptine	681.92	5	15.8	5.42	328.3	495.5
Cabergoline	213.7	0.6	0.9	0.8	56.2	22.4
Lisuride	64.6	0.3	0.7	0.99	4.6	3.5
Pergolide	850.9	31.6	25.7	3.9	58.9	475.6
Quinagolide^a^	5400	n.a.	0.5	3.2	n.a.	n.a.

Affinities are reported as inhibition constant [Ki] in nM [Ki Database-https://pdsp.unc.edu]

^a^Binding affinities of quinagolide are reported as dissociation constant [Kd] in nM-[Dopamine Receptors and Antipsychotic drugs in Health and Disease-Encyclopedia of Neuroscience, Larry R. Squire]

The clinical efficacy of DAs on the inhibition of hormone secretion and tumour growth in several diseases involving the hypothalamus-pituitary axis is a consequence of different mechanisms of action triggered by the activation of D2-like receptors, particularly of D2 receptor. The activation of D2 receptor with DAs has been demonstrated to be an effective mechanism for inhibiting pituitary hormones, mainly PRL, GH, TSH, and ACTH, secretion in rodent and human pituitary tumour primary cultures and cell lines [23, 61, 76–80], and for inhibiting proliferation and/or inducing apoptosis in rodent and human lactotroph, somatotroph, corticotroph and non-functional pituitary tumour primary cultures and/or cell lines [81–84]. Among DAs, BRC and CAB possess a potent dopaminergic activity, displaying high affinity for D2-like receptors but with the ability to bind also D1-like receptors, although CAB, characterized by a more potent selectivity and affinity and longer-acting activity on D2 receptor, demonstrated a greater efficacy compared to BRC in inducing PRL normalization and lactotroph tumour shrinkage [75].

The use of BRC or CAB have, in experimental setting, led to a better characterization of D2 receptor signalling pathways involved in hormone secretion and cell proliferation in lactotroph cells either in physiological or in pathological conditions, emphasising different properties of the two DAs in the activation of intracellular molecular pathways in lactotroph cell models. The PRL secretion inhibition by BRC is mainly related to the stimulation of K^+^ and sodium (Na^+^)-ATPase activity and the increase of cytosolic Ca^2+^ concentration, which further inhibit cAMP production as demonstrated in rodent pituitary lactotroph normal and tumour cells [85]. Conversely, the regulation of the PRL secretion by CAB is mainly related to MAPK pathways as demonstrated in a rodent pituitary somatotroph tumour cell line [54]. Interestingly, BRC and CAB show a different mechanism of action even in the regulation of cell proliferation as demonstrated in a rodent pituitary lactotroph tumour cell lines. Despite autophagy and apoptosis represent the main and common CAB-related and BRC-related molecular mechanisms, CAB more strongly activates autophagy, acting through the inactivation of AKT/mTOR pathway, whereas BRC more strongly activates apoptosis, increasing the expression of the zinc finger transcription factor early growth response protein 1 (EGR1) and the activation of MAPK pathway, both through the activation of ERK 1/2 and c-Jun N-terminal kinase (JNK) [82,86].

Nowadays, the guidelines for the treatment of hyperprolactinemia mainly recommend CAB as the treatment of choice to control circulating PRL levels and eventual lactotroph tumour growth, in case of PRL-secreting pituitary tumours [16]. Additionally, CAB is currently considered as a potential option for medical treatment of non-functional pituitary tumours [14] and GH-secreting pituitary tumours responsible for the development of gigantism or acromegaly [17], as well as in ACTH-secreting pituitary tumours responsible for the development of CD [3].

Figure 2B reports the intracellular mechanisms underlying autophagy and apoptosis triggered by D2 receptor (D2S and D2L) upon binding of DAs.

### Cushing’s syndrome

CS is characterized by a clinical syndrome, which is common, expect for few distinctions, to the three types of disease, including pituitary-dependent CS or CD, adrenal CS and ectopic CS [1, 2].

The clinical syndrome of CS usually includes moon face with facial plethora, cervical and supraclavicular fat depots, central obesity and proximal muscle atrophy with weakening of the limbs and general asthenia, skin thinning associated with purplish striae and/or acne and hirsutism, and susceptibility to diffuse bruising. CS is complicated with an increase in mortality and an impairment of the quality of life, due to the presence of several comorbidities including metabolic syndrome (visceral obesity, hypertension, hyperglycaemia and dyslipidaemia), cardiovascular disease with thrombosis diathesis, osteopenia or osteoporosis with increased risk of pathological fractures, neuropsychiatric disorders, and susceptibility to infections as well as reproductive and sexual dysfunction [1, 2]. In patients with CS, treatment aims at normalizing cortisol secretion, reverting the clinical picture, preventing comorbidities and systemic complications, and removing responsible tumour or control tumour growth, with the attempt of ensuring long-term disease control without recurrence [1, 2].

The first-line treatment for all types of CS is the surgical removal of the responsible tumour. However, although in the ACTH-independent forms of CS, the adrenal tumour is easily visualized and removed, in the ACTH-dependent forms of CS, the tumour can be hardly visualized or removed, making surgery unfeasible or ineffective and determining persistence or recurrence of hypercortisolism. In patients with CD, several second-line therapies are available, including repeat pituitary surgery, pituitary radiotherapy, adrenal surgery for rarely monolateral or more frequently bilateral adrenalectomy, and medical therapy; therefore, the treatment choice may be characterized by an individualized approach. Adrenal surgery for monolateral or bilateral adrenalectomy and medical therapy are potential options also for adrenal and ectopic CS. Nowadays, medical therapy is gaining importance in the management of CS, being potentially employed in different phases of the treatment algorithm, being potentially used to control hypecortisolism: (1) before surgery, as presurgical treatment; (2) after surgery, as adjuvant treatment in patients not cured by surgery, (3) after radiotherapy, as bridging treating while awaiting for the complete efficacy of radiotherapy in controlling cortisol secretion, or (4) as first-line treatment in patients not eligible for surgery; however, it still remains a palliative treatment which cannot cure the patient. Three types of medical treatment for CS have been developed, including the pituitary-directed drugs, which target the pituitary tumour inhibiting the ACTH secretion (for CD), the adrenal-directed drugs, which target the adrenal and inhibit the adrenal steroidogenesis (for all CS types), and glucocorticoid receptor-directed drugs, which act at peripheral levels, blocking the activity of glucocorticoid receptors (for all CS types). The totality of developed drugs is effective only in subsets of patients, being in the remaining cases ineffective or not tolerated, especially when used for long periods [3, 87]. In this context, the expression of DRs in the pituitary, adrenal or ectopic tumours or lesions causing CS, represents a potential target for treatment with DAs, which can be considered a generally well tolerated medical option.

### DRs expression in tumours responsible for Cushing’s syndrome

The expression of DRs in corticotroph pituitary tumours, cortisol-secreting adrenal tumours or lesions and corticotroph extra-pituitary tumours represents the prerequisite for a potential employment of DAs in the treatment of CS.

Regarding corticotroph pituitary tumours, in animal models, D2 receptor expression has been described either at messenger or protein level in percentage higher than 61% and nearly 43%, respectively, of canine corticotroph pituitary tumours [88, 89]. In humans, D2 receptor expression has been clearly demonstrated either at messenger or at protein levels in variable percentages between 37.5% and 83.3% and between 20% and 89.4%, respectively, according to the different techniques used for receptor detection [19–23, 62, 90]. The correspondence between messenger and protein levels, documented in a study using different techniques [23], suggests that the evaluation of D2 receptor messenger might be a surrogate of D2 receptor protein expression in human corticotroph pituitary tumours. Nevertheless, it should be kept in mind that D2S and D2L expression have been always investigated only at messenger levels due to the lack of available specific antibodies for the study of D2 receptor isoforms at protein levels. In the same study, D2S and D2L, have been documented, with only D2S present in 20%, only D2L in 40% and the two isoforms together in 40% of cases expressing D2 receptor, whereas D4 receptor was reported in 17% of cases, with absence of expression of the remaining DRs [23]. Notably, in a different study, the more frequent expression of D2L compared to D2S was confirmed, whereas the D4 receptor resulted not detectable [21], suggesting a potential variable expression of D4 receptors in corticotroph pituitary tumours. Few studies have investigated the existence of a potential correlation between D2 receptor expression and clinical picture. Indeed, no association between D2 receptor expression and tumour size or infrasellar and parasellar invasiveness, respectively evaluated by Knosp and Hardy classifications, has been reported [22]; interestingly, no differences were also reported in D2 receptor expression between microadenomas or macroadenomas or between presurgically uncontrolled and controlled patients [19]. This evidence seems to suggest that D2 receptor expression in corticotroph pituitary tumour is not influenced by the tumour biology or the degree of cortisol secretion under different medical treatments. However, classifying corticotroph pituitary tumours according with Wilson classification, non-invasive tumours presented relatively higher D2 receptor expression than invasive tumours [21]. Interestingly, silent and functional corticotroph pituitary tumours express D2 receptor with a similar prevalence [20].

Regarding adrenal tumour or lesions associated with CS, DRs expression has been scarcely evaluated. However, in one study in humans, the expression of the totality of DRs, except for the D3 receptor, has been documented at messenger levels in adrenal hyperplasia, with variable expression of the various DRs in different types of adrenocortical adenomas and carcinomas. Considering exclusively cortisol-secreting adrenocortical tumours, both adenomas and carcinomas expressed D2 receptor either at messenger or protein level**,** although benign tumours expressed both D2S and D2L whereas malignant tumours expressed D2L but not D2S [18].

Regarding corticotroph extra-pituitary tumours, DRs expression has been scarcely evaluated. In particular, in one study in humans, D2 receptor expression was described at protein level in five of six (83.3%) tumours including four lung, one thymic and one pancreatic tumour, responsible for ectopic CS, with the pancreatic tumour being the only negative case; in three of the lung tumours expressing D2 receptor, evaluated at messenger level, D2S was found in one case and D2L in all three cases, with additional D4 receptor in two cases and absence of the remaining DRs [24]. Notably, the expression of D2 receptor has been reported in series of neuroendocrine tumours, which also included some cases associated with ectopic CS [91, 92].

Table 2 summarizes the evidence of D2 receptor expression in tumours responsible for the different types of CS. Figure 3B shows some exemplary pictures of D2 receptor protein expression and the relative rate of expression in normal and pathological tissues involved in determining different types of CS.

**Table 2:** D2 receptor expression in tumours responsible for the different types of CS

	Protein expression	Messenger expression							
	Number of positive cases (percentage)	Number of positive cases (percentage)							References
	D2	D1	D2	D2L	D2S	D3	D4	D5	
Corticotoph pituitary tumours	5/6 (83.3%)	Not reported	3/8 (37.5%)	Not reported	Not reported	Not reported	Not reported	Not reported	Stefaneanu [62]
	15/20 (75%)	0/12 (0%)	10/12 (83.3%)	8/12 (66.7%)	6/12 (50%)	0/12 (0%)	2/12 (16.7%)	0/12 (0%)	Pivonello [23]
	not reported	Not reported	25/30 (83.3%)	78%	not reported	Not reported	0%	Not reported	de Bruin [21]
	1/5 (20%)	Not reported	Not reported	Not reported	Not reported	Not reported	Not reported	Not reported	Pawlikowski [90]
	17/19 (89.4%)	Not reported	Not reported	Not reported	Not reported	Not reported	Not reported	Not reported	Sickler [22]
Cortisol-secreting adrenocortical tumours	4/4(100%)	1/4 (25%)	4/4 (100%)	4/4 (100%)	2/4 (50%)	0/4 (0%)	4/4 (100%)	0/4 (0%)	Pivonello [18]
*Adenomas*	2/2(100%)	1/2 (50%)	2/2 (100%)	2/2 (100%)	2/2 (100%)	0/2 (0%)	2/2 (100%)	0/2 (0%)	
*Carcinomas*	2/2(100%)	0/2 (0%)	2/2 (100%)	2/2 (100%)	0/2 (0%)	0/2 (0%)	2/2 (100%)	0/2 (0%)	
Corticotroph ectopic tumours	5/6 (83.3%)	0/3 (0%)	3/3 (100%)	3/3 (100%)	1/3 (33.3%)	0/3 (0%)	2/3 (66.7%)	0/3 (0%)	Pivonello [24]
	3/3 (100%)	Not reported	Not reported	Not reported	Not reported	Not reported	Not reported	Not reported	Grossrubatscher [91]

### Preclinical functional studies with DAs in tumours responsible for Cushing’s syndrome

Few studies evaluated the effects of DAs in preclinical models of corticotroph pituitary tumour, cortisol-secreting adrenal tumours or lesions and corticotroph extra-pituitary tumours.

In AtT20, a D2 receptor expressing murine corticotroph cell line, which represents the most used preclinical model of pituitary corticotroph tumours, BRC did not reduce pro-opiomelanocortin (POMC), the ACTH/melanocortin precursor, expression and ACTH synthesis, but inhibited cell proliferation and induced apoptosis [77, 81, 93]. Conversely, an effect on hormone production was observed in canine corticotroph pituitary tumour primary cultures, where CAB inhibited the CRH-induced ACTH secretion [88]. Similarly, both BRC and CAB inhibited ACTH secretion in at least 50% of human corticotroph pituitary tumour primary cultures [19, 23]; importantly, a significant dose-dependent inhibition of ACTH secretion was found after both BRC and CAB administration only in primary cultures expressing D2 receptor, with ACTH response to DAs correlated with D2 receptor expression [23].

In NCI-H295R, a human adrenocortical carcinoma cell line producing aldosterone and cortisol, and representing a preclinical model of adrenal CS, D2 and D4 receptors were found to mediate the dopamine regulation of aldosterone, and not cortisol, production [70]. Moreover, in human adrenal hyperplasia cell primary cultures, a potentially more feasible model of adrenal CS, CAB, but not BRC, significantly inhibited both baseline and ACTH-stimulated aldosterone, but not cortisol production [18], suggesting that the expression of DRs in adrenal cortex might be mainly involved in the regulation of aldosterone rather than cortisol secretion. However, the two models actually resemble an adrenocortical malignant tumour or a non-tumoral lesion, respectively, both not completely resembling the lesions causing adrenal CS, mostly represented by adrenocortical benign tumours.

In CORL103, a small cell lung cancer cell line, which represents a potential model of corticotroph extra-pituitary tumours, BRC has been reported to inhibit the secretion of an ACTH precursor [77]. No data on CAB effects in experimental models of extra-pituitary tumours have been reported.

This evidence suggested a potential use of DAs in the treatment of CD and possibly ectopic CS, but probably not in adrenal CS. Indeed, clinical studies are presently available for CD and ectopic CS.

### Clinical studies with DAs in Cushing’s disease

Several clinical studies have investigated the effect of DAs, particularly BRC and CAB, in the treatment of CD, evaluating their efficacy in the inhibition of cortisol secretion and in the control of the corticotroph pituitary tumour.

Since 1976, the pioneering studies of Lamberts and co-workers have hypothesized DAs, particularly BRC, to be a potential treatment for CD. However, the greatest experience with DAs in the management of CD was accumulated in the last two decades, with the first translational experience reported in 2004 by Pivonello and co-workers, who focused on the more potent DA, CAB, and tested its efficacy on a relevant number of patients with CD.

BRC was the first DA historically used in the treatment of CD, displaying variable results [3, 94–99]. Indeed, in a study on 26 patients with CD, BRC, administered at a dosage of 1.25–30 mg/day for at least 3 weeks, induced remission, in terms of normalization of urinary or plasma glucocorticoids, in 42.3% of cases [97]. Moreover, a 50% remission, sporadically associated with tumour shrinkage, has been reported in a study including a small number of patients with CD treated with high BRC dosages (up to 55 mg/day) for a maximal period of 36 months [98]. Interestingly, treatment escape after an initial beneficial effect of BRC, transiently overcome by dose increase, was reported in a relevant subgroup, estimated to correspond at least to 33.3%, of patients after long-term treatment [99].

CAB is the most common DA used in the medical treatment of CD. A recent meta-analysis, including data of 124 CD patients, from six studies published from 2009 to 2017, showed that the proportion of patients achieving remission, in terms of normalization of urinary cortisol levels, after CAB therapy, for 1–105 months, was 34%, with a range from 20 to 40% [25, 26]. The presence of previous surgery, the treatment duration and the maximum CAB dose were identified as predictors for CD remission. Among patients who responded to CAB therapy, data on long-term treatment were available in 36 patients, with eight, corresponding to 22.2%, displaying treatment escape [25]. CAB treatment was associated with a good tolerance and a rate of severe adverse events of 5.6%; five patients developed adrenal insufficiency, with four requiring hydrocortisone treatment and one discontinuing treatment, whereas two patients had hypotension, inducing discontinuation [25]. On the basis of this evidence, the meta-analysis suggested that CAB therapy may be a reasonable alternative in the treatment of persistent or recurrent CD, potentially useful as second-line treatment after failure of pituitary surgery, or as first-line treatment as pre-surgical therapy or instead of surgical therapy in case of contraindications for or refusal of pituitary surgery [25].

In a pilot translational study, the effectiveness of CAB treatment was studied in 10 patients with CD, unsuccessfully treated by pituitary surgery [23]. The responsiveness to CAB treatment was evaluated according to changes in urinary cortisol excretion, with a decrease ≥ 50% considered a significant clinical response; in particular, patients who achieved a ≥ 50% decrease with normalization of urinary cortisol levels were considered full responders, whereas those who achieved a ≥ 50% decrease without normalization of urinary cortisol levels were considered partial responders, and those who achieved a < 50% decrease were considered non-responders. After a short-term (3 months) CAB treatment at a dosage of 1–3 mg/week, a significant inhibition of cortisol secretion was found in 60% of cases, with 40% considered full and 20% partial responders [23]. A trend for a significant association between CAB response and tumour D2 receptor expression was observed; indeed, the totality of responsive cases were associated with D2 receptor expression, whereas the totality of non-responsive cases were associated with absence of D2 receptor expression, with the exception of one case, displaying an absent or poor response despite the D2 receptor expression [23]. Moreover, among the six responsive cases, the four full responders expressed D2S except for one case expressing D2L associated with D4 receptor, whereas the two partial responders expressed D2L; the only non-responsive case expressing D2 receptor displayed D2 receptor expression documented at protein level without information on the messenger expression of D2 receptor and isoforms, and D4 receptors. The evidence reported in this pivotal translational study suggested a potential efficacy of CAB treatment with a good tolerance at medium–high dose in the management of CD.

In the following years, several studies have tested the efficacy and safety of CAB focusing mostly on the subgroup of patients with persistent CD after failure of pituitary surgery [27–31]. CAB, administered at a dosage of 0.5–7 mg/week, has shown remission rates, in terms of control of cortisol excess, of 25–40%; the cortisol reduction was accompanied by an improvement in the clinical picture, mainly hypertension and glucose intolerance, with an escape rate of 18.2–33.3% of patients with initial treatment response [27–31]. Particularly, in the first open-label prospective study, CAB was administered to 20 patients with CD unsuccessfully treated by pituitary surgery [27]. The responsiveness to CAB treatment was evaluated according to changes in urinary cortisol excretion, with complete normalization considered as a full response and a decrease ≥ 25% considered as a partial response at short-term evaluation; persistence of normal cortisol excretion was the only criterion to evaluate the response at long-term evaluation. After 3 months (short-term) of treatment at a dosage of 1–3 mg/week, 15 (75%) patients were responsive, particularly, seven (35%) patients were fully responders and eight (40%) partially responders. During the following period of treatment up to 12–24 months (long-term), at a dosage of 1–7 mg/week, five (33.3%) of 15 initially responsive patients showed a treatment escape, and two (10%) of the initial 20 patients starting treatment discontinued for hypotension and severe asthenia after 12 and 18 months of treatment, respectively, demonstrating that eight (53.3%) of 15 initially responsive patients continued to safely respond to CAB for a long-term period of treatment. Considering the initial population of 20 patients, the success rate of CAB treatment was 75% (15 patients) after 3 months, 50% (10 patients) after 12 months, and 40% (8 patients) after 24 months. The clinical picture improved in most responsive patients, in terms of symptoms and signs, mainly regarding muscle mass and strength and skin features, and comorbidities, mainly including hypertension, impairment of glucose metabolism and obesity, whereas it mildly improved, remained stable, or mildly worsened in non-responsive patients. A significant (> 25%) tumour shrinkage was observed in 50% of long-term responsive patients, whereas a stable tumour volume was found in the remaining responsive patients, with a slight increase in the only resistant patient who continued treatment for 12 months [27]. In a second retrospective study, CAB was administered to 30 patients with CD, including 27 patients with persistent CD after surgery and three naïve patients [28]. The responsiveness to CAB treatment was evaluated according to changes in urinary cortisol excretion, with complete normalization of urinary cortisol levels considered as a full response and a decrease of urinary cortisol levels to < 125% of the upper limit of normal but without complete normalization as a partial response. After 3–6 months (short-term) of treatment, at a dosage of 0.5–4 mg/week, 15 (50%) patients were responsive, with 11 (36.7%) patients fully responders and four (13.3%) partially responders. After 12–60 months (long-term) of treatment at a dosage of 0.5–6 mg/week, nine (30%) patients showed a persistent efficacy, maintaining a complete response. The urinary cortisol reduction was accompanied by a significant improvement in clinical picture, but an escape was observed in two (18.2%) of the 11 initially fully responsive patients, after 2 and 5 years, respectively [28].

CAB efficacy was also evaluated in smaller case series [29, 30]. Particularly, CAB was administered to 12 patients with CD unsuccessfully treated by pituitary surgery; after 6 months of treatment at a dosage of 1–3 mg/week, three (25%) patients had normalization, with the remaining nine (75%) displaying a reduction of 15–48.4%, of urinary cortisol levels, together with an improvement of clinical picture in the totality of cases [29]. Similarly, in another study, CAB was administered to six patients with persistent or recurrent CD or naïve; after 6 months of treatment at a dosage of 0.5–3 mg/week, two (33.3%) patients had normalization, whereas three (50%) reported a reduction of 21 to more than 30%, of urinary cortisol levels, together with an improvement of body mass index, waist circumference and blood pressure [30].

In a prospective, open-label, single-arm study, CAB was administered to 20 patients with persistent or recurrent CD [31]. The responsiveness to CAB treatment was evaluated according to changes in midnight and/or serum cortisol after low-dose dexamethasone. After 12 months of treatment, at a dosage of 1–5 mg/week, seven (35%) patients reported a normalization of midnight and/or post-dexamethasone cortisol levels; however, among these seven patients, five had received radiotherapy, which was considered responsible for the cortisol response in two patients maintaining cortisol normalization after CAB withdrawal. Therefore, with the exclusion of these two patients, five (27.8%) of 18 patients were considered responsive to CAB treatment. The clinical picture improved in terms of signs and symptoms, mainly including a clear reduction or disappearance of facial mooning and plethora in the responsive patients, and comorbidities, with an improvement in blood pressure and a substantial weight reduction in one patient. No significant change was noted in pituitary tumour size in both responsive and non-responsive patients [31].

More recently, in 2016, an additional study was published, with one important difference compared with the previous ones, namely the inclusion of a great majority of naïve patients [100]. Indeed, CAB was administered to 20 patients with CD, 19 naïve and only one with recurrent CD after pituitary surgery. After a short-term treatment of 6 weeks, at a dosage of 0.5–5 mg/week, no significant changes neither in median urinary and salivary cortisol levels nor in body weight, blood pressure, fasting glucose and HbA1c were reported. However, five (25%) of 20 patients showed more than 50% reduction, with two (10%) displaying normalization, of urinary cortisol levels, whereas two (10%) additional patients normalized and nearly normalized, despite a less than 50% reduction, urinary cortisol levels; therefore, four (20%) patients fully or nearly normalized urinary cortisol levels. The conclusions of the study suggested that short-term treatment with CAB may have a limited value in CD management [100]. Notably, this study, which included in the analysis almost exclusively naïve patients, evaluated efficacy after a very short treatment duration, probably too short to detect CAB effect in CD treatment.

Lastly, in 2017, a retrospective multicentre study reported the results of CAB treatment in 62 CD patients, with 53 receiving CAB monotherapy and nine receiving CAB as an add-on treatment with steroidogenesis inhibitors [32]. CAB monotherapy was administered to 53 CD patients, 44 with persistent/recurrent CD after pituitary surgery and nine naïve; 21 (39.6%) patients fully normalized urinary cortisol levels within 12 months at a dosage of 0.5–6 mg/week, and 12 (22.6%) patients, after a median of 32.5 months (range 19–105), at a dosage of 0.5–3.5 mg/week. Notably, 4 (7.5%) additional patients displayed a partial response to the treatment. The urinary cortisol reduction was accompanied by an improvement of clinical picture, particularly body weight, glucose metabolism and blood pressure. Treatment escape was reported in seven (38.9%) of the 18 initially responsive patients treated for more than 12 months, after a median treatment duration of 26 months. Treatment discontinuation was reported in eight (12.9%) of the entire cohort of 62 patients because of poor tolerance [32].

Table 3 summarizes clinical studies evaluating CAB monotherapy in CD patients.

**Table 3:** Clinical studies evaluating CAB monotherapy in CD patients

Study, year [References]	Type of study	N. Pts	Type of patients	Drug dose range (mg/week)	Follow-up M, m (range) (months)	Remission rate (*%*)	Escape (% of initially responsive)	Clinical picture improvement	Tumor shrinkage	Adverse events
Pivonello et al. 2009 [27]	Prospective, open-label, single arm	20	20 PS	1–7	M:21.6; m:24 (12–24)	40.0	33.3	Body mass index, blood pressure, glucose metabolism, muscle mass and strength, skin features	Yes	Dizziness, nausea, asthenia, hypotension
Godbout et al. 2010 [28]	Retrospective, single arm	30	27 PS; 3 N	0.5–6	M:37 (12–60)	30.0	18.2	Clinical symptoms and signs of CD	NE	Dizziness, nausea
Vilar et al. 2010 [29]	Prospective, open-label, single arm	12	12 PS	1–3	M:6, m:6	25.0	NE	Clinical symptoms of CD	NE	Dizziness, nausea
Barbot et al. 2014 [30]	Prospective, double arm with control	6	5 PS; 1 N	0.5–3	M:6, m:6	33.3	NE	Waist circumference, Body mass index, blood pressure	No	None
Lila et al. 2010 [31]	Prospective, open-label, single arm	18^a^	20 PS (5 RT)	1–5	M:12, m:12	27.8	NE	Body weight, blood pressure, facial mooning and plethora	No	None
Burman et al. 2016 [100]	Prospective, open-label, single arm	20	19 N; 1 PS	0.5–5	M:1.5, m:1.5	15.0	NE	No	NE	Dizziness, nausea, asthenia, visual hallucinations, constipation, mild gastrointestinal discomfort, loose stools, nasal congestion, change in personality
Ferriere et al. 2017 [32]	Retrospective, single arm	53	44 PS 9 N (15 RT)	0.5–6	m:32.5 (19–105)	23.0	38.9	Body weight, glycemic control and blood pressure	NE	Dizziness, nausea, asthenia, hypotension, dyspepsia, abdominal pain, muscle pain, alopecia and edema

*PS* previous pituitary surgery, *N* naïve, *RT* previous or concomitant radiotherapy, *NE* not evaluated, *M* mean, *m* median

^a^Excluding 2 patients who had received radiotherapy, which was considered responsible for their cortisol response

The efficacy of CAB has been evaluated in combination treatment with the steroidogenesis inhibitors, ketoconazole and metyrapone, as well as with the SRL pasireotide [29, 30, 32, 33]. The efficacy of CAB in combination with the steroidogenesis inhibitors ketoconazole or metyrapone, has been investigated in three independent studies, which reported a normalization of urinary cortisol levels in 68.8% (range 55.6–78.6%) patients, after 6–12 months of treatment [29, 30, 32]. Particularly, CAB treatment at a dosage of 0.5–3.5 mg/week induced remission in six (66.7%) of nine patients after 6 months, when associated with 200–400 mg/day of ketoconazole [29], in 11 (78.6%) of 14 patients, after 6 months, when associated with 200 mg/day of ketoconazole [30] and in five (55.6%) of nine patients during the first year, when associated with 600–1200 mg/day of ketoconazole or 3750–6000 mg/day of metyrapone [32]. Interestingly, the combined approach increased the treatment success as compared with monotherapy, representing an effective second-line treatment in patients with persistent CD after unsuccessful pituitary surgery. The efficacy of CAB in combination with the SRL pasireotide has been investigated in in two major studies [33, 34]. In one published study, 17 CD patients were included in an 80-day trial. The responsiveness to treatment was evaluated according to changes in urinary cortisol levels with complete normalization considered as a full response. The totality of patients started treatment with pasireotide at a dosage of 300–750 μg/day, with 5 (29.4%) patients displaying a full response. At day 28, in the 12 patients without normalization of urinary cortisol levels, CAB was added to pasireotide, at a dosage of 2–6 mg/week, with full response in additional four (23.5%) patients. At day 60, in the eight (47%) patients without normalization of urinary cortisol levels despite the combination therapy with pasireotide and CAB, the addition of ketoconazole (600 mg/day) induced full response in six (35.3%) additional patients, with a final success rate of 88.2% patients using single, double or triple therapy [33]. In another study, whose definitive data are not yet available but preliminary results have been presented in endocrinological meeting [34] and discuss in a recent review [4], 68 CD patients were included in a 35-weeks trial. The primary endpoint of this study was the proportion of patients achieving mean urinary cortisol normalization with pasireotide monotherapy (1200–1800 μg/day) or in combination with CAB (0.5–1 mg/day). All patients started treatment with pasireotide and CAB was added in patients who did not achieve biochemical control after 18 weeks of pasireotide monotherapy. After 35 weeks of treatment, 34 (50%) patients achieved mean urinary cortisol normalization, of which 17 (25%) achieved the goal with pasireotide in monotherapy whereas the remaining 17 (25%) required the combined therapy with CAB [34].

As far as safety profile was concerned, CAB was very well tolerated with rare adverse events, mainly including hypotension (0–10%) and asthenia (0–30%), after a prolonged period of treatment at the maximal dose of CAB, beyond dizziness and/or nausea (0–35%), often transiently present at the initial period of treatment [25, 27–32, 100]. Less common adverse events were vertigo (3.2%), constipation (2.4%) together with diarrhoea, oedema, nasal congestion, personality change and visual hallucination (0.8%) [25]. The increased risk of cardiac valve disease, reported at higher dosage used for neurological disorders, are less important at lower dosage used to treat CD [27–30].

Several case reports demonstrated that CAB induced normalization of cortisol and/or ACTH secretion, and/or improvement of clinical picture, and/or significant tumour shrinkage, until disappearance of the pituitary tumour, in different patients with corticotroph tumour progression after bilateral adrenalectomy, namely Nelson's syndrome [40, 41, 101], as well as in a patient with silent ACTH-positive macroadenoma [102], in a patient with functional ACTH-secreting macroadenoma [103], in different patients with functional ACTH-secreting microadenoma [104], as well as in an aberrant ACTH-secreting macroadenoma [105], in monotherapy. A successful treatment with CAB in combination with ketoconazole has been also described in another functional ACTH-secreting macroadenoma [106]. Notably, CAB has been shown to safely control cortisol excess during pregnancy in women with CD, being associated with an uncomplicated antenatal course and safe delivery [107, 108].

### Clinical studies with DAs in ectopic CS

The efficacy of CAB treatment has been reported in small series and sporadic cases of ectopic CS due to extra-pituitary corticotroph tumours. Particularly, in three patients with persistent ACTH-secreting lung carcinoids after surgery, which expressed D2 and in two cases even D4 receptors, normalization of urinary cortisol levels was reported in two (66.7%) patients, bearing a tumour expressing D2 and D4 receptor, after 3 months of treatment at a dosage of 3.5 mg/week, with a significant improvement in clinical picture, particularly blood pressure and glucose metabolism. However, persistent control was found in one case, with tumour expressing both D2S and D2L, whereas treatment escape was demonstrated in one case, with tumour expressing D2L, associated with re-worsening of clinical syndrome; notably, the non-responsive case had a tumour weakly expressing D2L without D4 receptor [24]. CAB has been also used in a case of occult source of ACTH secretion, thereafter, revealed as a polypoid lesion in the right sphenoidal sinus, showing normalization of urinary and salivary cortisol levels after 2 months of treatment at dosage of 3.5 mg/week, associated with normalization of blood pressure and menses [35].

The efficacy of CAB treatment in therapeutic sequence or in combination with SRLs and with steroidogenesis inhibitors has been also reported in few cases of ectopic CS. A case of ACTH-secreting lung carcinoid was managed after thoracic surgery, ketoconazole and aminoglutethimide, and bilateral adrenalectomy failures, with octreotide treatment at dosage of 20 mg/month for three years and, subsequently, CAB at dosage of 3.5 mg/week for 8 years, with the achievement of a marked reduction of ACTH levels, depigmentation and disappearance of the tumour mass detected by yearly lung computed tomography [39]. Some cases have also demonstrated the hypothesis that DAs and SRLs, in combination, may potentiate their actions in the management of ectopic CS. Particularly, a case of lung carcinoid tumour was long-term successfully managed, after surgery failure, with a 12-month combined treatment of CAB at dosage of 7 mg/week associated with 90 mg/month of SRL lanreotide [37]. Similarly, another more recent case report has confirmed this evidence describing the efficacy of CAB associated with the SRL octreotide in one ACTH-secreting lung carcinoid successfully and rapidly managed before surgery with a 2-week combined treatment of CAB at dosage of 1.5 mg/week associated with 0.05 mg/8 h of octreotide, leading to fast clinical improvement of blood pressure and glucose metabolism [38]. In a recent case series, CAB was also used at a dosage of 1.75–7 mg/week, essentially in therapeutic sequence and/or combination with steroidogenesis inhibitors in nine patients with severe CS of ectopic or occult origin. The effectiveness of CAB treatment after 5–29 months enabled rapid withdrawal of the steroidogenesis inhibitors and long-term control of the hypercortisolism in three (33.3%) cases [36]. Nevertheless, CAB in combination with steroidogenesis inhibitors failed to normalize the ACTH levels in five of the six cases, while the last case was difficult to judge based on the evolution of ACTH [36]. However, further studies on a larger number of patients are mandatory to confirm the usefulness of CAB alone or in combination treatment in the management of ectopic CS.

CAB has been shown to safely control cortisol in patients with CS during paediatric age [109, 110], suggesting that CAB may be a possible choice of treatment also for paediatric CS.

## Conclusions

In conclusion, the dopaminergic system controls and modulates the HPA axis in normal and pathological conditions. The DR expression and function have been found to promote inhibition of hormone secretion and/or cell proliferation in experimental models of corticotroph pituitary and ectopic tumours. The expression of DRs in pituitary and extra-pituitary tumours causing CS, together with experimental effects of DAs on hormone secretion and cell proliferation, suggested that DAs might represent a potential medical treatment for CS. Medical therapy has been shown to play a key role in the management of patients with CS over the last years, and nowadays represents an important alternative treatment approach. CAB is the most common DA used in the medical therapy of CS, since it has demonstrated a reasonable efficacy and a good safety. As off-label drug, CAB may be considered a medical option especially for persistent or recurrent CD, in several conditions, particularly, as adjuvant treatment after the failure of pituitary surgery, as bridge therapy while awaiting for the efficacy of pituitary radiotherapy, and even as first-line treatment occasionally before pituitary surgery, in case of long waiting and/or willing to improve clinical profile before the surgical approach, or instead of pituitary surgery, in case of refusal or contraindications for surgery, with a preference for mild hypercortisolism. Interestingly, the use of combined medical therapy approach of CAB with steroidogenesis inhibitors or SRLs has been reported to generally increase the likelihood of treatment success. Moreover, CAB has been demonstrated to display beneficial effects also in ectopic CS, either in monotherapy or in combination with SRLs or steroidogenesis inhibitors, although data are presently based on a limited number of experiences. Despite the positive evidence on the usefulness of treatment with CAB, further controlled studies on larger populations are needed to better evaluate the clinical advantages, in terms of efficacy and safety, of the treatment with CAB, and in general with DAs, in the management of the different forms of CS.
